# Early success of the COCOON trial: Preventing dermatologic adverse events in first-line EGFR-mutant NSCLC

**DOI:** 10.18632/oncoscience.648

**Published:** 2026-03-11

**Authors:** Bishal Tiwari, Asmita Koirala

**Affiliations:** ^1^Nassau University Medical Center, East Meadow, NY 11554, USA; ^2^Western Regional Hospital, Pokhara, Nepal

**Keywords:** EGFR-mutant non-small cell lung cancer, amivantamab, lazertinib, dermatologic adverse events, COCOON trial

## Abstract

Dermatologic adverse events (AEs) are a well-recognized complication of EGFR-targeted therapies, often emerging early during treatment and contributing to dose interruptions, patient discomfort, and reduced adherence. The combination of amivantamab, a bispecific EGFR-MET antibody, with lazertinib, a third-generation EGFR tyrosine kinase inhibitor, has demonstrated significant survival benefits in EGFR-mutant advanced non-small cell lung cancer (NSCLC). However, this therapeutic advancement brings with it an increased incidence of cutaneous toxicity. At the 2025 European Lung Cancer Congress, interim findings from the phase II COCOON trial were presented, offering timely insight into a proactive approach to managing these toxicities.

This commentary summarizes and contextualizes the COCOON study, which investigated whether a structured dermatologic prophylaxis regimen could mitigate moderate-to-severe skin AEs in patients receiving first-line amivantamab plus lazertinib. The prophylactic protocol included oral doxycycline or minocycline, ceramide-based moisturization, chlorhexidine nail care, and topical clindamycin, initiated at specified intervals. Compared to standard reactive care, this strategy halved the incidence of grade ≥2 dermatologic AEs (38.6% vs. 76.5%) and reduced grade ≥3 events and treatment discontinuations.

The COCOON results emphasize the clinical value of anticipating EGFR inhibitor-related toxicities through multidisciplinary supportive care. By implementing straightforward, low-cost interventions, clinicians can significantly improve tolerability and maintain dose intensity, maximizing therapeutic benefit. As this data informs future updates to clinical practice guidelines, it reinforces the need to integrate dermatologic prevention into first-line treatment planning for EGFR-mutant NSCLC. This commentary highlights COCOON’s relevance as a model for supportive care innovation in targeted oncology.

## INTRODUCTION

Epidermal growth factor receptor (EGFR) inhibitors have revolutionized the treatment of EGFR-mutant non-small cell lung cancer (NSCLC), but their use is frequently complicated by dermatologic adverse events (AEs) such as acneiform rash, pruritus, dry skin, and paronychia [1]. These cutaneous toxicities can affect over half of treated patients and typically appear early in the course of therapy, often within the first few weeks to months [1]. If not adequately managed, EGFR inhibitor-associated skin AEs can significantly impair patient quality of life and lead to dose interruptions or discontinuation of life-prolonging therapy. Traditionally, dermatologic AEs have been managed symptomatically with topical corticosteroids, emollients, and antibiotics once rash or other symptoms arise. However, accumulating evidence suggests that a proactive approach can mitigate these side effects.

Prophylactic skin treatments have been explored in earlier studies of EGFR-targeted therapies. A landmark randomized trial in metastatic colorectal cancer (the STEPP study) demonstrated that a pre-emptive skin regimen (including moisturizer, sunscreen, topical steroids, and doxycycline) significantly reduced the incidence of ≥grade 2 rash from 62% to 29% compared to reactive treatment [2]. Likewise, a 2016 meta-analysis by Petrelli et al. found that patients who began oral tetracycline antibiotics at the start of EGFR inhibitor therapy had markedly lower risk of skin rash; likely owing to tetracyclines’ anti-inflammatory properties, which are independent of their antimicrobial effects, prophylaxis halved the odds of developing rash (odds ratio ~0.53), and moderate-to-severe eruptions were reduced by ~70% [3]. In that analysis, the incidence of high-grade (≥grade 2) rash dropped from ~50% in control arms to ~24% with prophylactic antibiotics [4]. These findings established that proactive skin toxicity management—especially with tetracyclines—can significantly reduce EGFR inhibitor-induced rash without compromising anti-tumor efficacy. As the authors noted, “taking preemptive tetracyclines for several weeks at the start of anti-EGFR treatment can significantly reduce the incidence and severity of cutaneous acneiform rash” [4]. This strategy has since been adopted in practice for some EGFR inhibitors, such as cetuximab or erlotinib, to help patients avoid severe rash and remain on therapy.

Amivantamab is a bispecific antibody targeting EGFR and MET receptors, recently approved in combination with the third-generation EGFR tyrosine kinase inhibitor lazertinib for first-line treatment of EGFR-mutated advanced NSCLC [5]. The phase 3 MARIPOSA trial showed that first-line amivantamab plus lazertinib significantly improved progression-free and overall survival compared to the prior standard osimertinib, establishing the combination as a new frontline option [6]. At a median follow-up of 37.8 months in MARIPOSA, the amivantamab-lazertinib regimen reduced the risk of death by 25% (HR 0.75, *P* < 0.005) and yielded a projected median overall survival over one year longer than with osimertinib alone [6]. However, this greater efficacy came at the cost of increased dermatologic toxicity. Notably, patients receiving the amivantamab-lazertinib combination experienced higher rates of rash and nail inflammation than those on Osimertinib [7]. In the MARIPOSA safety analysis, most skin AEs (such as acneiform rash, dry skin, and paronychia) were observed early—often within the first 4 months on therapy—and were generally of low-grade but could accumulate or worsen with continuous treatment [1]. Real-world experience with amivantamab monotherapy (e.g., in EGFR exon 20 insertion patients) similarly reported nearly all patients developing dermatologic AEs, including rash (in ~78–89% of patients) and paronychia, with some experiencing painful fissures or scalp lesions [1]. Such toxicities not only necessitate supportive care but also risk premature discontinuation of an otherwise effective therapy. Therefore, as amivantamab plus lazertinib enters widespread first-line use, there is a pressing need for strategies to proactively manage dermatologic AEs and preserve patients’ ability to continue treatment.

Preclinical rationale and early clinical practice supported a proactive dermatologic regimen for the amivantamab-lazertinib combination. Based on prior EGFR TKI studies and small institutional experiences, oncologists hypothesized that a simple prophylactic regimen—including an oral tetracycline antibiotic to prevent acneiform rash, skin moisturizers to maintain barrier function, and antiseptic measures for nail beds—could significantly reduce the incidence of moderate-to-severe skin toxicities [1]. In fact, one academic center that treated initial patients with amivantamab recommended instituting skincare precautions before the first infusion: in a retrospective analysis, Basse et al. observed 100% of their amivantamab-treated patients developed rash and paronychia despite reactive management, and subsequently advocated for prophylactic doxycycline, aggressive moisturizing, and nail care starting 1–2 weeks prior to amivantamab initiation [1]. Such a proactive, multidisciplinary approach was expected to reduce the frequency of dermatologic events and their complications (e.g., superinfection of rash or nail beds), thereby improving patients’ quality of life and allowing them to remain on therapy longer.

Against this backdrop, the COCOON trial (NCT06120140) was designed to rigorously evaluate an enhanced dermatologic management protocol versus standard care in patients receiving first-line amivantamab plus lazertinib. Launched in late 2024, COCOON is a global phase II study and, to our knowledge, the first prospective trial in NSCLC to test a pre-emptive strategy for EGFR inhibitor skin toxicity. This research perspective, written as a conference-style summary, highlights the interim findings of COCOON on preventing moderate-to-severe dermatologic AEs in EGFR-mutant advanced NSCLC. We also contextualize these results with background literature on EGFR inhibitor skin toxicities and prior prophylactic interventions, and discuss the implications for integrating proactive dermatologic care into frontline management of EGFR-mutated lung cancer.

## PATIENTS AND METHODS

### Study design

COCOON (ClinicalTrials.gov NCT06120140) is an ongoing phase II, open-label, randomized trial evaluating enhanced versus standard dermatologic management in patients with untreated EGFR-mutant advanced NSCLC who receive amivantamab plus lazertinib. The trial enrolled approximately 200 patients globally with locally advanced or metastatic NSCLC harboring common EGFR mutations (exon 19 deletion or L858R) and no prior systemic therapy for advanced disease [8]. Key eligibility included ECOG performance status 0–1 and absence of uncontrolled comorbidities or contraindications to the prophylactic medications (such as tetracycline allergy) [8]. Participants were randomized 1:1 into two arms: Arm A received amivantamab-lazertinib with proactive dermatologic prophylaxis (the “COCOON” regimen), while Arm B received amivantamab-lazertinib with standard-of-care dermatologic management. Randomization was stratified by age (<65 vs. ≥65 years) and race (Asian vs. non-Asian) to ensure balance of these factors, which could potentially influence rash incidence [9]. The study is open-label given the nature of the intervention patients and providers could not be blinded to whether prophylactic skin treatments were being used.

### Treatment and interventions

All patients received the combination of amivantamab and lazertinib at standard doses for first-line therapy. Amivantamab was administered intravenously at 1050 mg (for weight ≤80 kg) or 1400 mg (>80 kg) weekly for the first 4 weeks, then every 2 weeks. Lazertinib was given orally at 240 mg once daily [9]. Beyond anti-cancer therapy, the arms differed in their skin care protocol as below.

### Arm A: Enhanced dermatologic prophylaxis (COCOON regimen)

Patients initiated a predefined prophylactic skincare regimen starting from cycle 1, day 1 of therapy. This regimen was developed to be simple, inexpensive, and easy to implement in any clinical setting [10]. It included: (a) Oral doxycycline or minocycline 100 mg twice daily for the first 12 weeks of treatment (an antibiotic known to reduce acneiform eruptions); (b) A 4% chlorhexidine antimicrobial wash applied daily to fingernails and toenails throughout therapy (to prevent nail bed infections and paronychia); (c) A ceramide-based, noncomedogenic moisturizer applied to the face and body at least once daily starting at treatment initiation and continued for 12 months (to maintain skin hydration and integrity); and (d) Starting at week 13, after completion of 12 weeks of systemic antibiotic, a 1% clindamycin topical lotion applied daily to the scalp (to specifically address any scalp folliculitis or lesions that can occur with prolonged EGFR inhibition). These measures were bundled as the “COCOON dermatologic management (DM) regimen.” In addition, Arm A patients received a smartphone-based digital health tool to track their adherence to the prophylactic interventions and prompt early reporting of any skin/nail symptoms. This remote monitoring helped ensure compliance and allowed investigators to intervene if signs of skin AEs emerged despite prophylaxis.

### Arm B: Standard-of-care dermatologic management

Patients in the control arm B received no mandatory prophylactic skin treatments at therapy start; instead, they were managed per routine practice. All patients were given general skin care recommendations (e.g., gentle skin cleansers, avoidance of sun exposure, use of sunscreen and emollients as needed), and physicians could institute reactive treatments for any dermatologic AEs that arose, such as prescribing topical or oral steroids, antibiotics, or dose modifications at their discretion. Essentially, Arm B represents the conventional reactive approach: treat skin toxicity once it appears and escalates, rather than preventing it upfront. It is important to note that both arms had equal access to reactive interventions for breakthrough skin AEs (ensuring ethical management of control patients), and the trial’s focus was on the addition of pre-emptive measures in Arm A.

Other supportive care was applied uniformly: for example, infusion-related reaction prophylaxis was given to all patients starting amivantamab (standard premedication with corticosteroids and antihistamines before the first infusion), and venous thromboembolism prophylaxis with oral anticoagulants was recommended during the first 4 months of therapy in both arms, following emerging data of elevated VTE risk with this combination. These measures were based on separate studies (e.g., the PALOMA studies for VTE prophylaxis and the SKIPPirr study for infusion reactions) and were incorporated into COCOON to maximize patient safety in both groups. Thus, the COCOON trial principally tested the impact of dermatologic prophylaxis while keeping other aspects of supportive care consistent.

### Endpoints and assessments

The primary endpoint of COCOON is the incidence of moderate-to-severe dermatologic adverse events of interest (DAEIs) within the first 12 weeks of treatment. “Dermatologic AEs of interest” included rash (acneiform rash and related terms), pruritus, skin dryness/fissures, eczema-like reactions, and nail fold inflammation (paronychia), which are the most common EGFR-related toxicities. In practice, this was defined as the proportion of patients who experienced any grade ≥2 dermatologic AE in the first 12 weeks from treatment start. Grade 2 AEs are those considered moderate in severity (e.g., rash covering 10–30% of body surface area or associated with symptoms), while grade 3 are severe (e.g., >30% BSA or causing significant pain or functional limitation), according to CTCAE v5.0 criteria. Secondary endpoints include the incidence of such AEs over the first 6 months; the incidence of severe (grade 3) dermatologic AEs; the number of distinct dermatologic AEs per patient; time to first onset of a grade 2 dermatologic AE; and the rates of dose interruptions, dose reductions, or treatment discontinuation due to dermatologic toxicity. Safety assessments were performed at regular intervals, with dermatologic examinations and AE grading at each clinic visit. An independent dermatology review committee adjudicated skin AE grading to ensure consistency. Quality-of-life (QoL) related to skin symptoms was also captured using dermatology-specific patient-reported outcomes, to be analyzed in a future report.

The trial was powered to detect a significant reduction in the 12-week ≥grade 2 DAEI rate with prophylaxis. An initial sample size of ~180 (90 per arm) was estimated to provide >90% power to detect a 50% relative risk reduction (from an expected ~60% incidence in standard care to ~30% with prophylaxis) at a two-sided α = 0.05. An interim analysis was pre-specified once roughly half the planned patients completed 12 weeks, to allow early assessment of efficacy. If the primary endpoint was met at interim, the protocol allowed for disclosure of results and potential adaptation of the trial (such as adding cohorts or refining prophylactic measures). Results of this interim analysis were presented at a major conference (European Lung Cancer Congress 2025) and are summarized below as the first report of COCOON’s findings.

## RESULTS

### Patient characteristics and treatment exposure

A total of 138 patients were included in the first interim analysis (Arm A, *n* = 70; Arm B, *n* = 68), reflecting those who had reached 12 weeks on therapy at the data cutoff. Baseline demographics were well balanced between the prophylaxis and control arms. The median age of participants was around 64 years (range 30–80), with 40% over age 65. Approximately 60% of patients were of Asian ethnicity (reflecting the high prevalence of EGFR-mutant NSCLC in Asian populations), and 40% were non-Asian; both arms had similar racial composition. Just over half of patients were female. All patients had EGFR exon 19 deletion or L858R-mutated tumors; about 55% had exon 19del and 45% L858R, with equal distribution between arms. At study entry, ~90% had metastatic (stage IV) disease and ~10% locally advanced IIIB/C; about 15% had treated brain metastases. ECOG performance status was 0 in ~45% and 1 in ~55%. No significant differences in these characteristics were noted between Arms A and B, and importantly there were no imbalances in known risk factors for skin toxicity (such as age or ethnicity). Adherence to the prophylactic regimen in Arm A was high: over 95% of patients took >90% of prescribed doxycycline doses in the first 3 months, and compliance with daily moisturizer and nail care was confirmed via the digital app and clinic visits. The median duration of therapy at the time of analysis was 5.2 months in Arm A and 5.0 months in Arm B, with all patients having completed at least 3 months on study.

### Primary endpoint - incidence of moderate-to-severe dermatologic AEs

**Figure 1:**
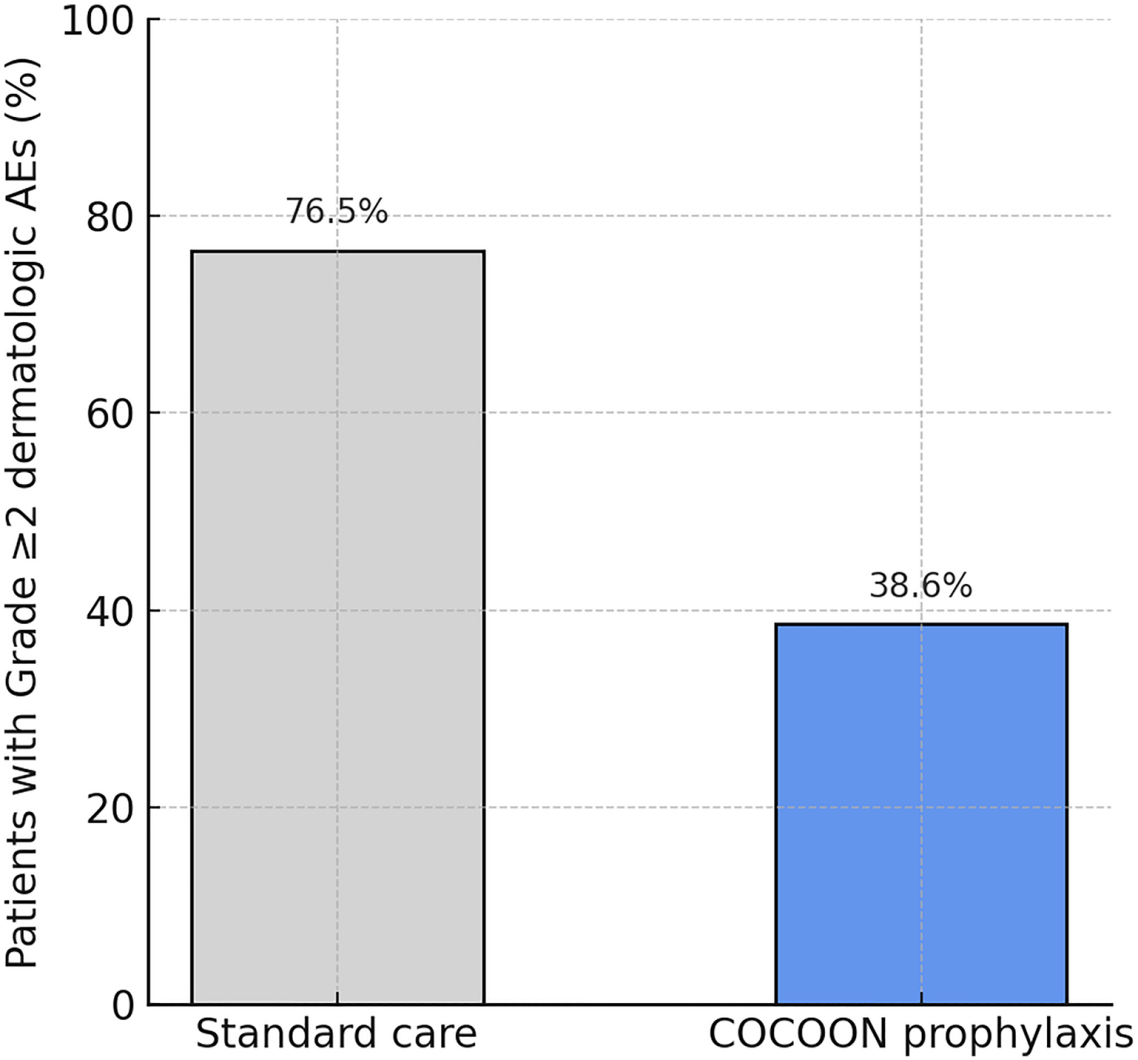
Incidence of moderate-to-severe ({greater than or equal to}Grade 2) dermatologic adverse events within the first 12 weeks of first-line amivantamab plus lazertinib, with and without prophylactic dermatologic management. The COCOON prophylaxis regimen halved the rate of significant skin AEs (38.6% vs. 76.5% with standard care). Bars show the percentage of patients in each arm experiencing any grade {greater than or equal to}2 dermatologic AE by week 12. Difference was statistically significant (*p* < 0.0001).

The COCOON trial met its primary endpoint at this interim analysis, demonstrating a statistically significant and clinically meaningful reduction in moderate-to-severe dermatologic adverse events with prophylactic management. In the first 12 weeks of treatment, only 38.6% of patients in the COCOON prophylaxis arm experienced any ≥grade 2 dermatologic AE, compared to 76.5% of patients receiving standard reactive management. This corresponds to roughly a 50% absolute reduction in risk (and a two-fold relative decrease) with the prophylactic regimen (Figure 1). The difference was highly significant (*p* < 0.0001), exceeding the trial’s efficacy threshold. Stated differently, three out of four patients on standard care developed at least moderate skin toxicity by 3 months, versus only about one in three patients on the COCOON regimen—a remarkable improvement. The calculated odds ratio was approximately 0.19 (95% CI: 0.09–0.41) in favor of the COCOON arm, indicating an 81% reduction in the odds of developing a ≥grade 2 dermatologic event. This magnitude of effect is rarely seen in supportive care interventions and underscores the efficacy of the prophylactic approach. Notably, the onset of skin AEs was also delayed in the prophylaxis group: among those who did get rash or related AEs, the median time to first grade 2 event was significantly longer in Arm A (not reached by 12 weeks, with many patients never developing moderate rash) compared to a median of ~6–8 weeks in Arm B (*p* < 0.001, data not shown).

Importantly, the benefit of prophylaxis was observed across all key subgroups analyzed. In prespecified subgroup analyses by age and race, the prophylactic regimen consistently reduced ≥grade 2 dermatologic AE rates compared to control, with no subgroup showing a diminished effect. For example, both younger patients (<65) and older patients (≥65) saw roughly a two-fold reduction in risk, and both Asian and non-Asian patients derived comparable benefit (data presented at ELCC 2025). This suggests the COCOON intervention is broadly effective regardless of patient demographics. Benefit was also evident across both major EGFR mutation types (ex19del and L858R) and was not influenced by performance status or gender. Thus, no particular subset of patients failed to benefit from prophylactic management; it appears universally helpful in this population.

Beyond the composite endpoint, prophylaxis markedly reduced the severity and multiplicity of skin AEs. The incidence of severe (grade 3) dermatologic AEs was cut by more than half, from 8.8% in the standard arm to 4.3% (3 of 70 patients) with COCOON prophylaxis. Grade 3 rash occurred in 4.4% (3 of 68 patients) on standard care and 1.4% (1 of 70) on prophylaxis. Furthermore, patients in Arm A were much less likely to experience multiple concurrent skin AEs. By 3 months, 18% of patients on standard care had suffered two or more distinct moderate/severe dermatologic AEs (for example, a significant rash and a severe nail infection), whereas only 6% of those on prophylaxis did so—a three-fold reduction. In summary, the COCOON regimen not only lowered the overall chance of any troublesome skin toxicity but also largely prevented the worst toxicities and the scenario of patients being “hit with” more than one dermatologic problem at the same time.

### Specific dermatologic AEs and affected sites

**Figure 2:**
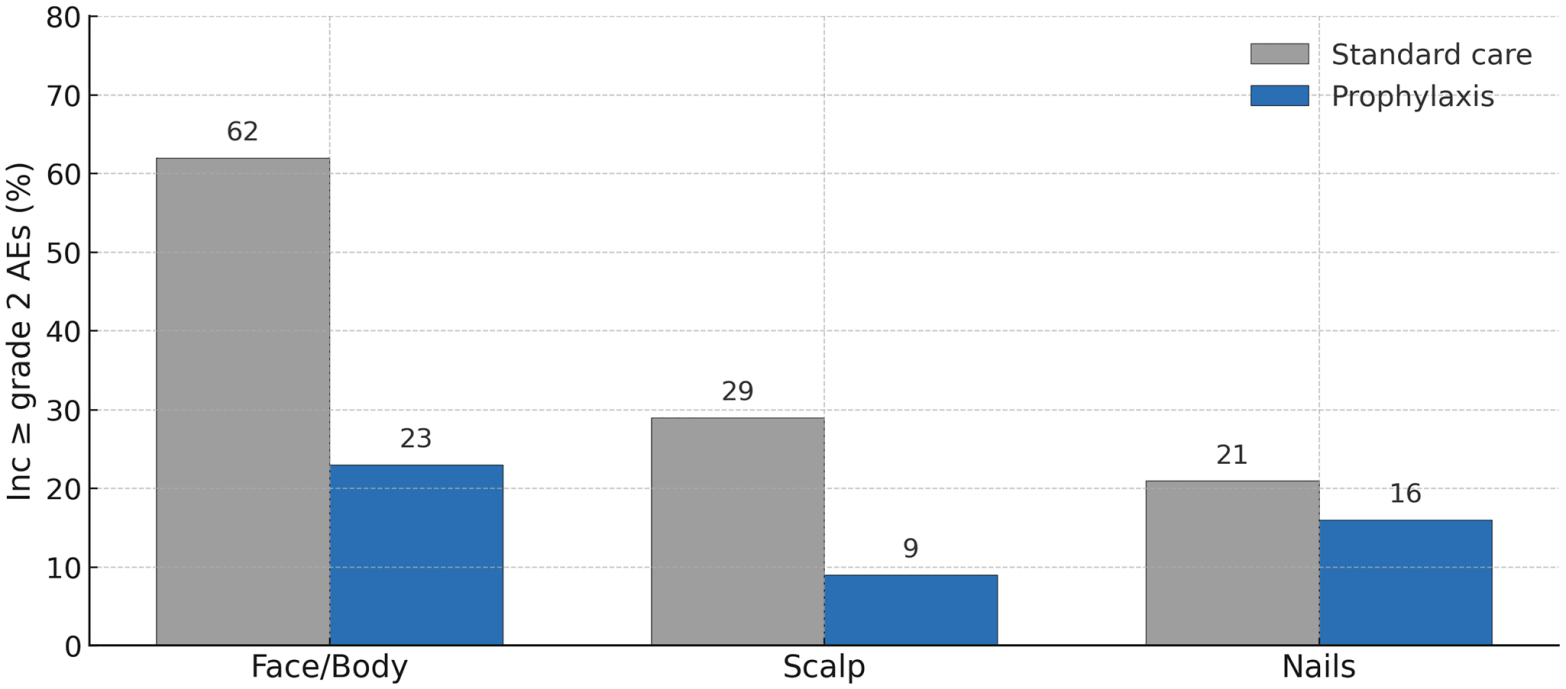
Incidence of moderate-to-severe dermatologic adverse events by category and body site during the first 12 weeks of treatment. Prophylactic dermatologic management (blue bars) dramatically reduced the occurrence of significant rash on the face/body and scalp, and slightly reduced nail inflammation, compared to standard reactive care (gray bars). Each bar represents the percentage of patients withgrade ≥2 AEs in that category (e.g., acneiform rash on face/body, scalp AEs, or paronychia). Prophylaxis conferred a 63% reduction in face/body rash and a 69% reduction in scalp AEs. All differences shown favored the COCOON prophylaxis arm.

The dermatologic AEs observed were in line with expectations for EGFR-directed therapy, with rash (acneiform eruption), dry skin, pruritus, and paronychia being the most common events in both arms. However, their frequency and distribution were substantially altered by prophylaxis. The incidence of ≥grade 2 AEs by anatomical site/manifestation in each arm is detailed in Figure 2. On the face and torso (trunk)—the typical locations of EGFR inhibitor acneiform rash—moderate-to-severe rash occurred in 62% of patients with standard management, versus only 23% with prophylaxis. Thus, the majority of patients on standard care developed noticeable rash on the face/body, whereas fewer than one-quarter did so when pre-emptively treated (a 63% relative reduction). Similarly, scalp involvement (which can manifest as painful folliculitis, pustules, or scabbing lesions in hair-bearing skin) was dramatically reduced: ≥grade 2 scalp AEs occurred in 29% of controls but just 9% of the prophylaxis arm. This >3-fold reduction in moderate/severe scalp toxicity is particularly noteworthy, as scalp lesions have been reported as one of the more difficult complications of amivantamab (sometimes leading to bleeding or infection of the scalp). Proactive use of topical clindamycin after week 12, combined with the systemic antibiotic earlier, likely helped protect the scalp follicles from intense inflammation once the combination therapy had been administered for a few months.

## TREATMENT MODIFICATIONS AND SAFETY

One of the most crucial implications of reducing dermatologic toxicity is the ability for patients to tolerate and continue cancer therapy without dose reduction or breaks. In the first 3 months, dose interruptions due to any AEs (primarily rash or infusion reactions) occurred in 34% of patients under standard care versus only 16% in the prophylaxis arm. Dose reductions of amivantamab or lazertinib (for toxicity) were required in 19% of standard arm patients but only 7% of those on prophylaxis. Focusing specifically on dermatologic causes, there was a three-fold reduction in treatment discontinuations due to skin AEs: in Arm B, 3 patients (4.4%) discontinued amivantamab–lazertinib permanently because of intolerable rash or skin toxicity, whereas only 1 patient (1.4%) in Arm A did so. When considering all causes, the overall discontinuation rates were low and not significantly different (since some patients in either arm discontinued for unrelated reasons or disease progression), but the nearly 70% drop in skin-related dropouts with prophylaxis is clinically meaningful. In short, the COCOON intervention allowed more patients to stay on the full, planned doses of amivantamab and lazertinib during the critical first-line treatment period. This was reflected in a higher proportion of patients remaining on therapy at 3 and 6 months in the prophylaxis arm (though long-term follow-up is needed to see if this translates to efficacy differences).

No new safety concerns emerged from adding the prophylactic measures. The tetracycline antibiotic (doxycycline or minocycline) was generally well tolerated; a few patients reported mild photosensitivity or upset stomach, but no grade ≥3 events attributable to antibiotics occurred. There were no cases of C. difficile infection or other serious sequelae of antibiotic use. The moisturizer and topical clindamycin caused no adverse effects aside from rare complaints of mild skin oiliness. Chlorhexidine nail solution was likewise benign; one patient had minor skin irritation from it, resolved by using it every other day. Importantly, the prophylactic regimen did not mask any serious conditions; for instance, no cases of drug reaction or other rashes went unrecognized. All skin AEs that did arise were appropriately identified and managed. There was some concern that prophylaxis might obscure an early rash that correlates with treatment efficacy (since historically rash severity has correlated with response to EGFR TKIs), but in this trial prophylaxis did not appear to reduce the anti-tumor effectiveness of therapy. By 12 weeks, the overall response rate to amivantamab–lazertinib was identical in both arms (~80%, per investigator assessment), indicating that preventing rash did not blunt the cancer treatment’s activity. In fact, maintaining full dose intensity likely optimizes efficacy.

## DISCUSSION

The COCOON trial provides solid evidence that proactive dermatologic care should be incorporated into the first-line management of EGFR-mutated NSCLC patients receiving amivantamab plus lazertinib. The concept of preemptive side-effect management is not new—as it has been explored with EGFR inhibitors in other cancers—but COCOON is the first study to validate this approach in the lung cancer setting with modern EGFR-targeted agents. The ~50% reduction in moderate-to-severe skin AEs observed is remarkably consistent with prior data on tetracycline prophylaxis (e.g., Petrelli et al.’s meta-analysis), reinforcing that these inexpensive measures can drastically alter the toxicity profile of EGFR inhibition.

From a clinical perspective, these results are practice-changing. Dermatologic toxicities from EGFR TKIs and antibodies have long been undermined as they are not life-threatening per se, but they cause considerable discomfort and can erode patient confidence in treatment. By intervening early, COCOON demonstrated improved tolerability of amivantamab–lazertinib without compromising efficacy. Ensuring patients can stay on the full dose of this combination likely helps maximize its benefit. It is notable that MARIPOSA showed a survival advantage over osimertinib despite higher toxicity; with better supportive care as shown here, the net clinical benefit of the combination could be even greater. As Dr. Nicolas Girard, who presented the COCOON data, aptly stated, managing these side effects proactively “clearly is significantly prolonging the overall survival of patients,” since patients who tolerate therapy can continue it longer and derive its full benefit.

The findings also refine our understanding of managing specific toxicities. The greater than three-fold reduction in moderate-to-severe scalp AEs (9% vs. 29%) is a valuable insight, as these can be particularly challenging. This suggests that the regimen’s approach—systemic antibiotics followed by targeted topical therapy is highly effective for this site. The modest reduction in nail toxicities (16% vs. 21%) is also encouraging, though it highlights that paronychia remains an area for further improvement, likely requiring a combination of antiseptic and mechanical preventive measures.

The COCOON results build upon decades of experience with other EGFR inhibitors. While proactive strategies were shown to be effective for first-generation agents like cetuximab and erlotinib, their adoption has been inconsistent. The clear, prospective, and dramatic results from COCOON for a modern, highly effective combination therapy should reinvigorate this practice. The proactive regimen is not only more effective than the reactive management in the control arm but also provides a standardized framework that can be easily adopted, moving beyond the variable “standard care” that often leads to suboptimal outcomes. For newer agents like osimertinib, which have a lower incidence of severe rash, such intensive prophylaxis may not be necessary for all patients, but the principles from COCOON can inform a risk-stratified approach.

Another discussion point is whether prophylaxis might reduce the correlation between rash and treatment efficacy. In EGFR TKIs, there has been an observed phenomenon that patients who develop rash often have better tumor responses (rash as a pharmacodynamic marker of EGFR inhibition) [4]. Some practitioners worry that preventing rash might mean under-treating the patient, but COCOON’s data argue against that. The anti-tumor efficacy (response rate at 12 weeks) was equivalent with prophylaxis, and as noted, maintaining full dose likely outweighs any hypothetical loss of a rash-response correlation. Prophylaxis simply reduces a side effect, not drug levels or activity. It is analogous to using growth factors for chemotherapy-induced neutropenia; we do not assume that supporting blood counts will reduce chemotherapy efficacy.

One limitation is that the data reported are from a pre-planned interim analysis. Longer follow-up will be important to assess the durability of the benefit and to see if the curves of skin AE incidence remain separated at 6 or 12 months. Another consideration is generalizability; while the trial focused on amivantamab + lazertinib, its principles could likely apply to other rash-inducing EGFR inhibitors. Finally, COCOON’s positive outcome has opened additional investigations. The trial protocol is being amended to incorporate a single-arm cohort evaluating subcutaneous administration of amivantamab with the prophylactic regimen. Subcutaneous amivantamab has shown non-inferior efficacy and reduced infusion reactions [11]. It will be valuable to see if this delivery route impacts skin AEs; it might not directly reduce rash, but if SC allows for a lower C_max (maximum serum concentration) or different pharmacokinetics, rash severity could differ.

## CONCLUSIONS

The COCOON trial’s early findings demonstrate that a pre-emptive dermatologic management strategy can dramatically reduce the incidence and severity of skin and nail toxicities in EGFR-mutant NSCLC patients receiving first-line amivantamab plus lazertinib. By halving the rate of moderate-to-severe rash and associated AEs, the prophylactic “COCOON” regimen (doxycycline, topical clindamycin, chlorhexidine nail care, and moisturizer) helps patients stay on therapy longer with improved quality of life. These results validate years of prior anecdotal and observational evidence suggesting benefits of prophylactic tetracyclines and skin care for EGFR inhibitor patients. Implementing this regimen in routine practice is expected to become a new standard of care alongside the amivantamab–lazertinib combination. Looking ahead, the full dataset from COCOON will shed light on longer-term outcomes and refine best practices. Nonetheless, even this interim analysis represents a significant and timely contribution to the lung cancer field. It empowers clinicians to manage targeted therapy side effects proactively, ensuring that patients can derive maximal benefit from life-extending treatments.
